# Association between *PSCA, TNF-α, PARP1 *and *TP53* Gene Polymorphisms and Gastric Cancer Susceptibility in the Brazilian Population

**DOI:** 10.31557/APJCP.2020.21.1.43

**Published:** 2020

**Authors:** Roberto Nery Dantas, Augusto Monteiro de Souza, Sylvia Satomi Takeno, Paulo Kassab, Carlos Alberto Malheiros, Eleonidas Moura Lima

**Affiliations:** 1 *Laboratory of Molecular and Structural Biology Oncogenetics, LBMEO, *; 3 *Postgraduation Program in Cellular and Molecular Biology,*; 4 * Department of Molecular Biology, Federal University of Paraiba, João Pessoa - PB, *; 2 *Postgraduation Program in Health Sciences, Santa Casa de São Paulo Medical Sciences Faculty, Sao Paulo - SP, Brazil. *

**Keywords:** Gastric cancer, single nucleotide polymorphism, genotyping, molecular markers

## Abstract

**Objectives:**

To evaluate the association of allelic and genotypic frequencies of *PSCA (rs2976392)*, *TNF-α (rs1800629)*, *PARP1 (rs1136410)* and *TP53 (rs368771578) *SNPs with GC susceptibility in a Brazilian population.

**Materials and Methods:**

This is a retrospective study, which included 102 paraffin-embedded adenocarcinoma tissue samples > 5 years of obtention, with 204 alleles for each studied SNP. Other 102 healthy tissue samples were included as controls. For analysis, the genotyping method Dideoxy Single Allele-Specific – PCR was used. Statistical analysis was performed with the Bioestat software 5.3, determining Hardy-Weinberg’s equilibrium for the genotypic frequencies p-values < 0.05 were considered significant.

**Results:**

*PSCA (rs2976392) *and *TNF-α (rs1800629) *SNPs were associated with GC in the analyzed samples (X^2^=10.3/102 and p<0.001/0.00001, respectively). *TNF-α (rs1800629) *SNP presented also a statistically significant relationship between its genotypes and the morphological pattern (intestinal/diffuse) (p<0.032). However, *PARP1 (rs1136410) *and *TP53 (rs368771578)* SNPs were in Hardy-Weinberg’s equilibrium and, therefore, were not significantly associated with GC in these samples (X^2^=0.73/2.89 and p<0.39/0.08).

**Conclusions:**

*PSCA (rs2976392)* and *TNF-α (rs1800629) *SNPs are potential molecular markers of susceptibility to GC development. *PARP1 (rs1136410)* and* TP53 (rs368771578) *SNPs were not associated with the risk of GC development.

## Introduction

Oncology molecular studies have enabled assistance in the diagnosis and therapeutic counseling, accelerating the application of the knowledge of cancer biology to the clinical practices. Gastric cancer (GC) is an important public health problem and the World Health Organization estimates 27 million new cases of this neoplasm by 2030. GC is the second leading cause of death in the Brazilian population, preceded only by lung cancer (Ferlay et al., 2015). Its lethality rate is higher than other neoplasms, such as colon, breast and prostate (Jemal et al., 2011).

The molecular events involved in gastric carcinogenesis are still poorly understood, and have revealed a wide variety of genetic, dietary, socioeconomic, and lifestyle aspects (Nagini, 2012). The genetic predisposition can be investigated by several variants, including single nucleotide polymorphisms (SNP), that play an important role in carcinogenesis and can be risk factors acting through environmental effect modulation and promoting cellular instability (Yin et al., 2009). SNPs can alter biological properties of the encoded protein and affect the levels of gene expression. In recent studies, the association of important specific genes (*PSCA, TNF-α, PARP1* and *TP53*) and the risk of developing GC was reported (Lu et al., 2010; Zhu et al., 2014; Hu et al., 2014; Zhang et al., 2013).

The prostate stem cell antigen *(PSCA)* gene has tumor suppressor characteristics and inhibits cell production and/or death induction activities. Individuals with low PSCA promoter activity are especially susceptible to diffuse gastric cancer (Yoshida et al., 2010). TNF-α is a proinflammatory cytokine that especially mediates host defense, with ability to induce the expression of various gene products (Tahara et al., 2011). TNF-α plays an important role in the onset and development of GC, and its SNPs are associated with GC risk (Zhang et al., 2017). Poly (ADP-ribose) polymerase-1 (PARP1) enzyme gene products are important proteins associated with cancer risk. They catalyze the base excision repair step in single-strand breaks, key to the deoxyribonucleic acid (DNA) repair process, and are needed for supporting repair proteins recruitment, such as XRCC1 (Feng and Koh, 2013). Another tumor suppression gene, *TP53 *regulates a wide range of functions in response to DNA damage, such as apoptosis and senescence (Liu et al., 2016). *TP53* is a central regulator of cell proliferation and death, restricting cell growth when genomic irregularities are present (Ortiz, 2018).

Studies aim to describe the association of certain SNPs with different behaviors of cancer cells in different types of tumors in an attempt to determine molecular markers (Lubbe et al., 2012; Yang et al., 2014). This study aims to evaluate the behavior of the gene *SNPs PSCA (rs2976392), TNFα (rs1800629), PARP1 (rs1136410) *and *TP53 (rs368771578)* as risk factors for GC susceptibility. 

## Materials and Methods

This is a retrospective study which relied on samples (more than 5 years of obtention) from the pathology anatomy dataset from both Hospital Napoleão Laureano and Hospital Universitario Lauro Wanderley - Federal University of Paraiba - Joao Pessoa-PB, Brazil. A total of 102 paraffin-embedded tissue samples with histopathological diagnosis of gastric adenocarcinoma were analyzed and 102 matched samples without GC of paraffin tissue were analyzed. This study was approved by the local Institutional Ethics Committee, registered under the license number CAAE: 39152214.6.0000.5183.

*DNA extraction*

Samples went through DNA extraction at LBMEO in accordance to the method described by Shang Rong-Shi et al (Shi et al., 2002), modified. The isolated genomic DNA was quantified with the NanoDrop™ 2000c Spectrophotometer (Thermo Fisher Scientific) and stored at -20ºC.

*Dideoxy single allele-specific PCR (DSASP) Method*

The DSASP method is based on chain termination inhibitors, dideoxynucleoside, established by the method of Sanger et al. The DSASP was previously validated by the Allele-Specific PCR-ASP method, as described by Lima et al (Lima et al., 2015). This genotyping method comprises four specific stages: I – SNP selection; oligonucleotide delineation (primer and complementary sequence); choice of the specific dideoxynucleoside to be incorporated into the SNP allele position; II – Asymmetric PCR of the chosen dideoxynucleoside; III - Hybridization reaction between the complementary sequence and the asymmetric PCR product; IV - Melting curve analysis by qPCR, developed and validated by Lima et al., (2015).

In order to genotype *PSCA (rs2976392), TNFα (rs1800629), PARP1 (rs1136410) *and *TP53 (rs368771578) *SNPs by the DSASP method, an asymmetric PCR was performed on each specific SNP, through the complementary sequence and dideoxynucleoside incorporation. The oligonucleotides were obtained by in silico validation (GeneRunner Software).

*In Silico validation*

The primers used by DSASP for *PSCA (rs2976392), TNFα (rs1800629), PARP1 (rs1136410) and TP53 (rs368771578)* SNPs were projected based on the Ensembl Genome bank through the GeneRunner software (Table 1) to evaluate the melting temperature (Tm), the secondary structure formation and the amplified fragment size. 

*Asymmetric PCR conditions*

Asymmetric PCR was performed in a final volume of 25μL, containing 200 μM dNTP (dATP, dCTP, dTTP and ddGTP); MgCl 22.0 mM; 20 ng/μL DNA, 200 pM primer and 1U AmpliTaq Gold (Life Technologies - Carlsbad, CA). PCR conditions for single stranded DNA asymmetric amplification were as follows: a 3-minute pre-denaturation (95°C); 80 cycles of three steps (20s denaturation at 95°C; annealing at 60°C for 45s and a 30s extension at 72°C), with a final 5-minute extension at 72°C. 

*Hybridization conditions*

The PCR amplification product went through hybridization under the following conditions: 200 pM of the complementary sequence at 4°C for 10 minutes.

*Melting Curve analysis*

The melting curve was analyzed to determine the Tm, performed by the real-time PCR equipment 7500 Fast Time-Time PCR System (Life Technologies - Carlsbad, CA), following these parameters: preheating from 25°C to 95°C for 1 min; reaching 60°C for 5 min and then gradual heating (1°C/min) to a temperature of 95°C for 5 min. For the analysis of the melting curve, SYBR Green Mix (Life Technologies - Carlsbad, CA) was used.

*Statistical Analysis*

The allelic frequencies and the genotypic distributions of the case/control samples were obtained by the Hardy-Weinberg equilibrium model. The association analysis was performed by the chi-square test and Fisher’s exact test, using the statistical program BioEstat 5.3, where p-values <0.05 were considered statistically significant for all tests.

**Table 1:** Genes and Their Respective Polymorphisms, as Well as Specific Primers and Complementary Sequences for Each Polymorphism,Amplified by the DSASP Method

Gene	SNP	Primers / Complementary Sequences
*PSCA*	rs2976392	5’-CTATTAATCTTTCTGGCCATCTGTCCGCA -3’
	(G>A)	5’-TSGAAGGAMAACAGCACRYAGATGCGGACAGTGGCCAGAAAGATTAATAG-3’
*TNFα*	rs1800629	5’-AAATGGAGGCAATAGGTTTTGAGGGGCA-3’
	(A>G)	5’-TCCYCATGCCCCTCAAAACCTATTGCCTCCATTT-3’
*PARP1*	rs1136410	5’-CTCGATGTCCAGCAGGTTGTCAAGC-3’
	(G>A)	5’­-CAAGGYGGAAATGCTTGACAACCTGCTGGACATCGAG-3’
*TP53*	rs368771578	5’-CCAGAATGCAAGAAGCCCAGANGGAAA-3’
	(G>A)	5’-TACCAGGGCAGCYACGGTTTCCNTCTGGGCTTCTTGCATTCTGG-3’

DSASP, Dideoxy Single Allele-Specific PCR; SNP, single nucleotide polymorphism.

**Table 2:** SNP Genotypic Distribution and Allelic Frequency among Genes from Gastric Cancer Samples and Controls

Gene (SNP)	Genotypic distribution	Genotypic distribution	Allelic frequency		p-value
	Case (%)	Control (%)			
*PSCA*	GG 11	GG 19	Ref	Variant	
*(rs2976392)*	AG 66	AG 50	A	G	0.001
	Reference AA 25	AA 33	0.57	0.43	
*TNF-α*	AA 28	AA 53			
*(rs1800629)*	AG 00	AG 41	G	A	0.001
	Reference GG 74	GG 08	0.73	0.27	
*PARP1*	GG 00	GG 00			
*(rs1136410)*	AG 16	AG 15	A	G	0.39
	Reference AA 86	AA 87	0.92	0.08	
*TP53*	GG 74	GG 72			
*(rs368771578)*	AG 23	AG 27	A	G	0.08
	Reference AA 05	AA 03	0.16	0.84	

SNP, single nucleotide polymorphism.

**Figure 1:**
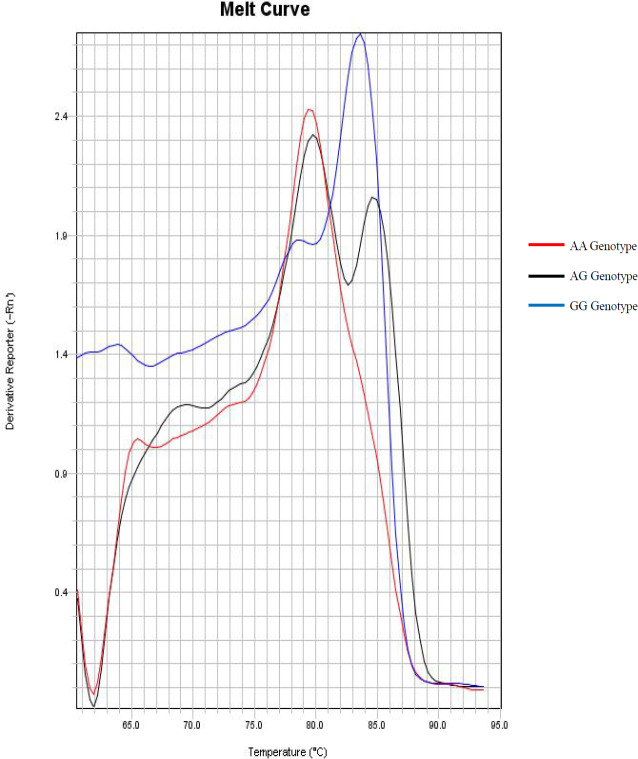
Melting Curves Related to AA, AG and GG Genotypes from PSCA (rs2976392) SNP. These Tm represent the double DNA fragments obtained by the DSASP technique

**Table 3:** Demographic and Histopathological Data Associated with TNF-α (rs1800629) SNP Genotypic Variant

	TNF-α (rs1800629) SNP				
	Total (n)	Genotype	Genotype	Genotype	p-value
		A/A	A/G	G/G	
Gender					
Male	67	21	0	46	
Female	35	7	0	28	0.2517
Age range (years old)					
≤ 60	45	12	0	33	
> 60	57	16	0	41	1
Anatomical location					
Antrum	48	14	0	34	
Body	36	9	0	27	
Fundus	15	4	0	11	
Cardia	3	1	0	2	0.9789
Morphological pattern					
Intestinal	71	24	0	47	
Diffuse	31	4	0	27	0.032

SNP, single nucleotide polymorphism.

**Figure 2:**
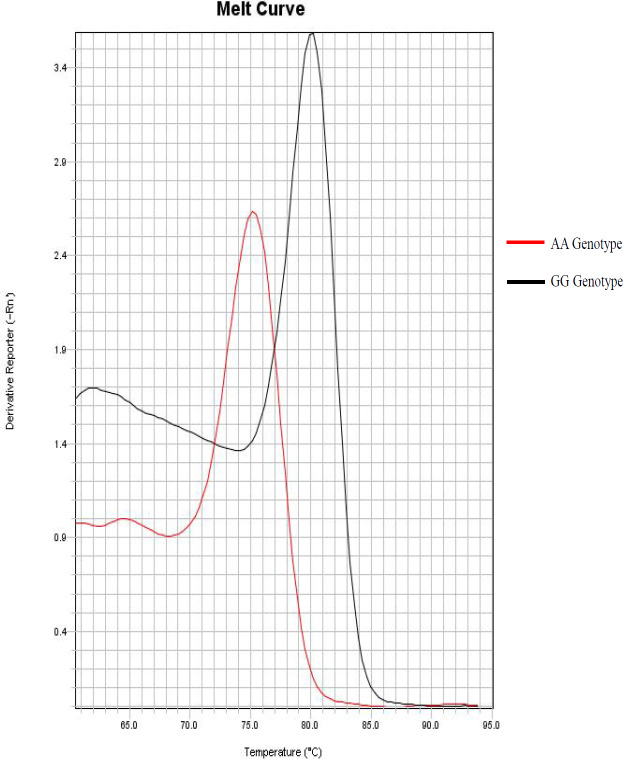
Melting Curves Related to AA and GG Genotypes from TNF-α (rs1800629) SNP. These Tm represent the double DNA fragments obtained by the DSASP technique

## Results

Clinical-histological data were analyzed in case-control samples, with age and sex matching of 1:1 with controls without gastric cancer. Of the analyzed GC patients, 65.68% were male. Participants mean age was 60.62 (28-84) years old. Regarding location, antrum tumors were prevalent (47.05%), followed by body (35.5%), fundus (14.7%) and cardia (2.95%). Considering the morphological pattern, the intestinal type was present in 69.6%, while the diffuse type in 30.4%. 

*SNP distribution and Cancer risk association*

A total of 102 adenocarcinoma tissue samples were analyzed, with 204 alleles for each studied SNP. Of the four studied SNPs, two were in Hardy-Weinberg’s imbalance (PSCA and TNF-α SNPs) (Table 2). 

PSCA (rs2976392) SNP genotypic frequencies were 10.79% (n=11) G/G; 64.70% (n=66) A/G and 24.31% (n=25) A/A. The polymorphic allele (G) frequency corresponds to 43% of the studied samples. The observed Tm for allele A ranged from 77 to 78ºC, meanwhile Tm for allele G ranged from 83 to 84ºC (Figure 1).

*TNF-α (rs1800629) *SNP genotypic frequencies were 27.45% (n=28) A/A; 0% (n=0) A/G and 72.55% (n=74) G/G. The polymorphic allele (A) frequency corresponds to 27.45%. The observed Tm for allele A ranged from 75 to 76ºC, while the G allele ranged its Tm from 80 to 82ºC (Figure 2).

After analyzing the association of *TNF-α (rs1800629) *SNP genotypes with other variables such as gender, age, tumor location and morphological pattern, our results suggested a statistically significant relationship only between the genotypes and the morphological pattern (p = 0.032) (Table 3).

*PARP1 (rs1136410)* and *TP53 (rs368771578) *SNPs were in Hardy-Weinberg’s equilibrium.* PARP1 (rs1136410) *SNP genotypic frequencies were 0% (n=0) A/A [ALA]/[ALA]; 15.69% (n=16) A/G [ALA]/[VAL] and 86% (n=84.31) G/G [VAL]/[VAL]. The polymorphic allele (A) frequency corresponds to 8%. The observed Tm for allele A ranged from 79 to 80ºC and Tm for allele G ranged from 86 to 88ºC. 

*TP53 (rs368771578)* SNP genotypic frequencies were 72.55% (n=74) G/G [HIS/HIS]; 22.55% (n=23) G/A [HIS/TYR]; and 4.90% (n=05) A/A [TYR/TYR]. The polymorphic allele (G) frequency corresponds to 84% of the studied samples. The observed Tm for allele A ranged from 73 to 75ºC, while Tm for allele G ranged from 79 to 81º.

## Discussion

Cancer is a multifactorial disease resulting from the interaction of genetic and environmental factors. Polymorphic loci are well studied in an attempt to find possible markers for prevention, diagnosis and treatment, or to help elucidate the molecular pathways involved in its pathogenesis. In this study, two of the analysed SNPs (PSCA and TNF-α) were significantly associated with GC risk, while the other two *(PARP1* and* TP53*) did not reach significance.

The *PSCA (rs2976392)* SNP was associated with GC risk, and its presence may correspond to a possible molecular marker for GC development. Studies report that the PSCA protein is related to some types of cancer such as: prostate, pancreas, esophagus and stomach (Qiao and Feng, 2012). Recently, a meta-analysis demonstrated that of the *PSCA rs2976392 G>A* polymorphism is associated with increased cancer risk, especially for gastric cancer and bladder cancer (Gu et al., 2015). In accordance, a meta-analysis performed with 16,792 subjects (9,738 GC cases and 7,054 controls), suggested this* PSCA *SNP was significantly associated with increased risk of GC, both diffuse and intestinal, among Chinese, Japanese and Korean individuals (Qiao and Feng, 2012).

The *TNF-α (rs1800629) *SNP was also associated with GC risk in our sample. When we analyzed the association of the SNP’s genotypes with the tumor’s morphological patterns, there was statistical significance (p=0.032). Many studies have linked this SNP with GC risk, promoting changes in transcriptional levels and affecting the secretion of protein (Xu et al., 2017). It has additionally been implicated in raising susceptibility to cervical and breast cancers (Ding et al., 2012; Fang et al., 2010; Xu et al., 2017). In 2014, 11 case/control studies with 7,427 patients were included in a meta-analysis which suggested its association with increased GC risk in Caucasians (Zhu et al., 2014).

*PARP1 (rs1136410)* and *TP53 (rs368771578)* SNPs were not associated with the risk of developing GC in our population. To date, about 1,066 *PARP1* SNPs play important roles in the DNA repair system in human carcinogenesis (Hua et al., 2014; Ang and Tan, 2017). Our results are similar to Hua et al findings in a meta-analysis with 43 studies, consisting of 17,351 cases and 22,401 controls (Hua et al., 2014). To better understand the role of *PARP1 (rs1136410)* SNP with GC susceptibility, further studies, especially in other populations, are required. Despite of the fact that our study did not show association of the *TP53 (rs368771578)* SNP with GC, several studies have shown evidence of significant differences in the biochemical properties of the TP53 protein as a function of the polymorphic alleles (Kim and Lozano, 2018).

Regarding the tumors’ morphological patterns in general, we observed a predominance of Lauren’s intestinal subtype (62.9%), which is the most frequent and depends directly on environmental factors, being associated with the presence of precancerous lesions, such as chronic gastritis, gastric atrophy, intestinal metaplasia and dysplasia (Huntsman et al., 2001). On the other hand, the diffuse pattern presented the worst predictors of poor prognosis: presence of perineural infiltrate, moderate to severe desmoplasia, lymph node metastasis and diagnosis in more advanced staging. For this subtype, mean age at diagnosis was 56.7 years, while the intestinal subtype was 62.3 years (Huntsman et al., 2001).

In our sample, males were predominant (65.68%), with mean age at diagnosis of 60.62 years. In a Brazilian study conducted by INCA in 2015, there were 9,132 deaths from GC in men (64.4%, mean age 61.7 years) and 5,132 in women. The prevalence of men corresponded to national and global estimates (Ferlay et al., 2015; Guedes et al., 2014).

Despite our findings, this is a small single center study. Larger studies considering these four studied SNPs are necessary, including a wider number of samples from different populations in order to better understand their role regarding gastric adenocarcinoma susceptibility.

In conclusion, studies of carcinogenesis have changed with the ability to detect SNPs. In this study, *PSCA (rs2976392)* and *TNF-α (rs1800629) *SNPs were significantly associated with GC risk, while *PARP1 (rs1136410)* and* TP53 (rs368771578) *SNPs were not. SNPs may be used as molecular markers for mapping and identifying genes that cause cancer. This understanding of molecular genetics in gastric carcinogenesis can have a major impact on the development of new target therapies, allowing treatment improvement and tumor resistance reduction.
